# Surface Modification of Poly(lactic acid) Fabrics with Plasma Pretreatment and Chitosan/Siloxane Polyesters Coating for Color Strength Improvement

**DOI:** 10.3390/polym9080371

**Published:** 2017-08-18

**Authors:** Chee-Chan Wang, Li-Huei Lin, Chi-Wu Chen, Yu-Chun Lo

**Affiliations:** Department of Cosmetic Science, Vanung University, 1, Van Nung Road, Taoyuan 32056, Taiwan; ccwang@mail.vnu.edu.tw (C.-C.W.); cwchen@mail.vnu.edu.tw (C.-W.C.); ac0212036@mail.vnu.edu.tw (Y.-C.L.)

**Keywords:** chitosan, siloxane, plasma, poly(lactic acid) fiber, dyeing

## Abstract

As people in the 21st century become increasingly environmentally aware, environmentally friendly products have come into focus. As such, environmentally friendly textiles and eco-textiles have become an international trend in research and development. Poly(lactic acid) fiber, which is biodegradable, holds much promise, but it is difficult to deep dye. This study used chitosan, succine anhydride, siloxane, and polyethylene glycol to produce a series of chitosan/siloxane polyesters that have a hydrophilic component (chitosan) and a hydrophobic component (siloxane), and this chitosan/siloxane polyester can be coated on poly(lactic acid) fiber, which we had subjected to Argon plasma treatment to increase their antimicrobial properties and to increase the fibers dyeing efficiency. The study shows that, after the surface plasma treatment, longer PEG chain lengths resulted in higher K/S values. This result suggests that the surface plasma pretreatment and chitosan/siloxane polyesters coating showed that lower ∆E values result in more leveling dyeing of poly(lactic acid) fiber.

## 1. Introduction

In the textile industry, a number of chemical reagents are used in conventional chemical processes. Many of these chemicals are toxic to humans and are not easily degraded in the environment. Eco-friendly processes are continuously being explored to substitute for toxic chemicals. Chitosan, an important and available biopolymer, has attracted more attention because of its biodegradability, biocompatibility [1,2], antimicrobial activity and non-toxicity [3]. Chitosan is a polysaccharide derived from glucosamine. It is a multifunctional, biocompatible, and environmentally friendly carbohydrate polymer, has low toxicity, and does not cause the body to create antibodies against it [4]. It has biological activity; can lower levels of cholesterol, blood lipids, and blood pressure; and enhance the immune response. It is biodegradable and can form a film or a gel [5]. It can be used in wound dressings, gauze patches, surgical sutures, antibacterial deodorant fabric, foods, immobilized enzymes, cosmetics [6], fruit juice clarifiers, fruit preservatives, and wastewater treatment agents. Chitosan is widely used in agriculture, medicine, foods, chemical engineering, environmental protection, and as an agent for improving the dyeability of fabric [7,8].

The hydrophobic structure of poly(lactic acid) allows it to be commonly used in dispersed dyes, but disperse dyes do not bind to poly(lactic acid) fibers well [9]. That is, they do not stain well, and the color does not hold fast, resulting in a large amount of dye remaining in dye baths and creating waste and environmental pollution, which limit the use of poly(lactic acid) fiber in the textile industry. In view of the surface modification of poly(lactic acid) fiber, the use of supercritical CO_2_ dyeing and other methods to improve the use of polyesters in improving the dyeing performance have been reported from a few studies [10,11,12], and this study will be done to address the problem with dyeing poly(lactic acid) fibers.

According to the literature [13,14], the glass transition temperature of poly(lactic acid) fiber is approximately 58 °C. In dyeing, the dye generally begins to stain the fabric around this temperature; as the temperature increases, the dye stains the fiber better. When the temperature reaches 110 °C, the dyeing rate reaches its maximum. In today’s industry, generally fibers are first spun and then dyed. Poly(lactic acid) fiber is first spun into yarn, and then dyed. However, because of the compact structure of the yarn, complete entry of the dye into the fiber is difficult, creating an uneven finish [15]. Because of the low affinity with which the dye binds, producing a deep color is impossible. If the fibers undergo dyeing before further processing, the fibers form into a stiff bundle, making them difficult to spin.

Schreiber et al. [16] was based on the avail-ability of thermodynamic interaction data for probes with *N*-Tetracosane (*N*-C_24_), di-*N*-octyl phthalate (DOP), and poly(dimethylsi1oxane). Self-consistent parameters are obtained for interactions between the probes and the pure stationary phases, and also those within the binary stationary phases. Su et al. [17] found a combination of Flory–Krigbaum theory and Yamakawa theory can be applied to both symmetric and moderately asymmetric ternary systems. From the light scattering results, the Flory–Huggins interaction parameter between polystyrene and poly(methyl methacrylate) was determined to be 0.009 ± 0.001 at 30 °C. Bhattacharyya et al. [18] showed that poly(vinyl ester)s and polyacrylates blends whose small molecular weight analogs exhibit exothermic mixing are miscible, whereas hydrogenated monomers are not always the proper analogs for vinyl polymers for the prediction of the interaction energy.

Plasma technology can save energy, water, time, dyestuff and finishing auxiliaries, and reduce wastewater pollution compared to conventional treatment techniques [16]. Plasma technology has proven to intensify dyeing rates of textile polymers improving the diffusion of dye molecules into the fibers, enhance color fastness and wash resistance of fabrics, increase adhesion of coatings, and modify the wettability of fabrics [17,18]. Plasma treatment has great practical value for the development of new functional fibers such as hydrophilic [19], dark-colored, shrink-proof, noncorrosive, and fireproof fibers. Plasma surface treatment is a low-temperature plasma application technique. The plasma processing system uses electrons to bombard gas molecules, causing ionization and division and producing electrically neutral molecular fragmentsl that is, high-energy free radicals. The ions in the plasma, either alone or in combination, then react on the surface of the material to produce the necessary reaction.

The plasma-treated polymer material surface may have a cross-linked structure or may have new functional groups. Hollahan et al. [20] treated the surface of a polymer with NH_3_ or N_2_-H_2_ mixtures and found that the plasma-treated polymer has an -NH_2_ group on the surface. Khoddami et al. [21] treated fabric with plasma polymerization technology to increase the melting point of the fabric and make it more water-resistant and softer. Fiber friction decreased and elasticity increased. They created easy-to-care textiles and wrinkle-free fabric. It not only retains the shape of the clothing, but also requires minimal ironing. Wrinkle-free textiles are characterized by good appearance, good wrinkle resistance, good recovery, resistance to mechanical washing, dimensional stability, anti-fouling properties, water resistance, minimum ironing requirements, and overall comfort. During plasma treatment, a fine mesh smaller than the dye molecules forms on the surface of the material, which suppresses detachment and fading of the dye.

This study prepares polyethylene glycol of different molecular weights (2000, 4000, 6000 and 8000 Daltons) into chitosan and siloxane to combine chitosan and siloxane (which have no interfaces) and create a series of chitosan/siloxane polyesters. Chitosan/siloxane-type compounds are coated onto poly(lactic acid) fabrics and treated with argon gas. After the plasma treatment, we analyzed the properties of the resulting fabric, including the molecular structure of the fabric Fourier Transform Infrared Spectroscopy (FTIR). The properties of fabrics containing chitosan/siloxane polyesters were also tested by Scanning Electron Microscopy (SEM). High-Resolution X-ray Photoelectron Spectroscopy (HRXPS) was done to investigate the surface morphology and roughness of chitosan/siloxane polyesters, as well as the surface and hydrophobicity of the fabric after coating in order to seek the best way in which the surface roughness of the fabric can improve the dyeing efficiency. In this study, we also investigated the antibacterial properties of chitosan/siloxane compounds coated onto the fabric.

## 2. Experimental

### 2.1. Materials

Chitosan was obtained from ACROS (Morris, JN, USA), which has a deacetylation degree of 85% and average molecular weight of 1.5 × 10^5^ Daltons. Commercial-grade, silanol-terminated polydimethylsiloxane (MW3400) was supplied by Dow Corning (Midland, MI, USA). Reagent-grade succinyl anhydride and polyethylene glycols (MW2000, 4000, 6000, and 8000) were purchased from Hayashi Pure Chemical (Osaka, Japan). The knitted poly(lactic acid) fibers were obtained from Unique Way International Inc. (Taipei, Taiwan) with a weight of 220 g/m^2^ (6.5 oz/yd^2^). The fabrics were made of both filament and staple fiber yarns. The yarn sizes of the filament and the staple were 90 denier and 25 Ne, respectively.

### 2.2. Synthesis

**Scheme 1:**
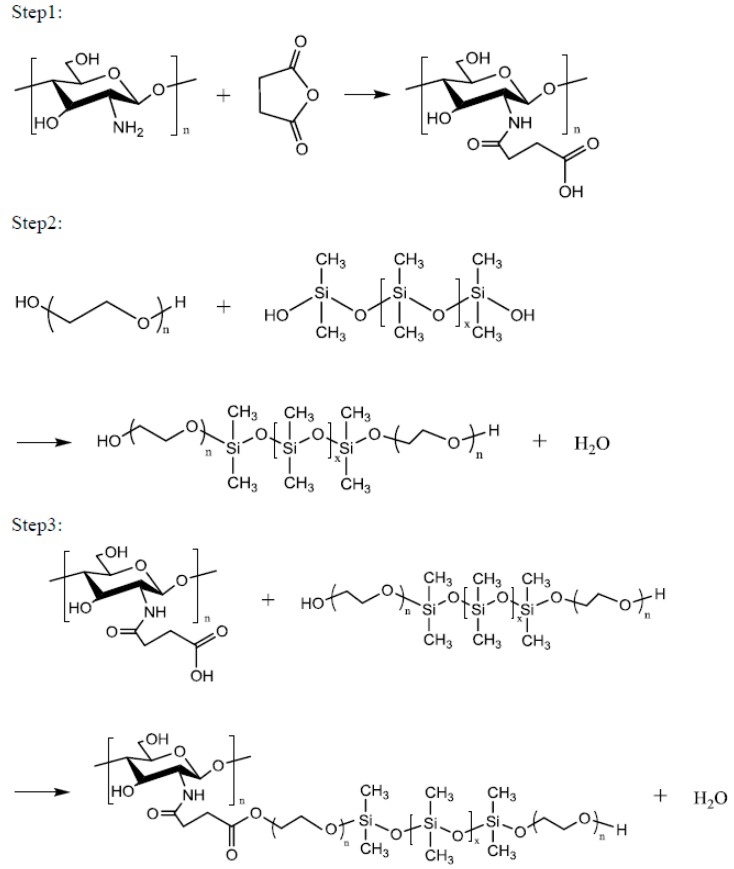
Synthesis of Chitosan/Siloxane polyesters.

Chitosan (1 mol) in 2% aqueous acetic acid and 1 mol of succinic anhydride were placed in a four-necked reaction flask equipped with a stir bar and a thermometer. Subsequently, 1 g of catalyst was added and the mixture was slowly heated to 50–70 °C. After 4 h of reaction at this temperature, impurities were removed via vacuum filtration, and the product was dried under vacuum at 50 °C to obtain the Step 1 product. One mole of polyethylene glycol (PEG2000, PEG4000, PEG6000 or PEG8000), 1 mol of siloxane, and 1 g of catalytic titanium isopropoxide were reacted at a temperature of 160 °C for 6 h to obtain the Step 2 product. One mole of the Step 1 product and 1 mol of the Step 2 product were reacted at 100–120 °C for 8 h to obtain a series of environment-friendly chitosan/siloxane polyesters (Scheme 1).

### 2.3. Analysis

A Fourier transform infrared (FTIR) spectrometer (Perkin Elmer Cetus Instruments, Norwalk, CT, USA) was used to monitor the spectral transmittance or absorbance of the chitosan/siloxane polyesters. High-resolution X-ray photoelectron spectroscopy (HRXPS, ULVAC-PHI Inc., Chigasaki, Japan) was performed using an ULVAC PHI Quantera SXM spectrometer(ULVAC-PHI Inc., Chigasaki, Japan), operated at 15 kV and 24.9 W with an energy resolution of 0.1 eV, and monochromated Al Kα radiation. The surface morphologies of the poly(lactic acid) fabrics were visualized using a Hitachi S-3000 N scanning electron microscope (SEM, Hitachi, Osaka, Japan).

### 2.4. Surface Plasma Treatment

Chitosan/siloxane polyesters with different PEG chain lengths (PEG2000, PEG4000, PEG6000 and PEG8000) were coated onto the fabric, and the antibacterial properties and changes in fabric dyeing properties (K/S test) between the plasma treated and non-plasma treated fabrics were compared.

First, argon was introduced and the partial pressure was adjusted to 1–1.5 atm. The system’s power and cooling system was opened, and the samples were individually placed on the reactor electrode plate. The vacuum was switched, and when the system pressure reached 5 mTorr, the reaction gas (NH_3_, Ar, and O_2_) was introduced. The pressure valve and flowmeter were adjusted to bring the system to the reaction conditions (pressure of 100 mTorr). The reaction time was set to 120 s each time. The plasma power was adjusted to 40 W.

### 2.5. Antibacterial Properties

The poly(lactic acid) fabric was cut into a standard 2.8 cm diameter circular piece. The test sample (5 g) was added to the cloth and coated evenly. The cloth was then dried at 60 °C in an oven and then placed on a sterile table and irradiated with UV light to sterilize it.

Transfer 10 μm *Staphylococcus aureus* culture and 5 mL of Luria–Bertani (LB) medium into a sterilized culture tube (180 mm), and then store the test tube in a shaking incubator at 37 °C for 16–18 h (keep the test tube slanted). Add 20 mL of LB to an Erlenmeyer flask, and then add 200 μm of the *S. aureus* culture and vortex. Transfer 1 mL of LB into a quartz tube and 1 mL of the bacterial mixture in an Erlenmeyer flask to another quartz tube. Use the LB medium as a blank to calibrate the spectrophotometer, and measure the OD of the bacteria mixture in the Erlenmeyer flask. When the OD value reaches 0.18, store the Erlenmeyer flask in a shaking incubator for 30 min to allow the bacteria to grow naturally. Repeat until the OD value reaches 1.8. Take two small, sterilized plates and place the control gauze in one and the poly(lactic acid) fabric in the other. Add 0.4 mL of the bacterial culture with OD value of 1.8 to the cloth, and let it stand at room temperature for 24 h.

Take several sterilized plates. Complete the LB drug preparation in a 100 mL beaker, add agar, and then pour into a bottle. Cover the bottle with aluminum foil and then sterilize for 20 min. Store the sample for 24 h at room temperature and then mix it with 4 mL of PBS (phosphate-buffered saline). Prepare a rack of microcentrifuge tubes (0.5 mL), add 0.9 μm of PBS to each, and then add 100 μm of the sample to produce 10-, 100-, 1000-, 10,000- and 100,000-fold dilutions. Sterilize and then place the rack in a sterile hood. Pour about 25 mL of LB agar into the culture plate, turn on the UV light, and wait for the agar to solidify. After it solidifies, flip the plate over, and label with sample and name. After labeling, add 100 μL of bacteria to the plate, and evenly disperse it. Seal the plate and incubate at 37 °C for 24 h, and then record the number of colonies.

### 2.6. Dyeing

A rapid laboratory dyeing machine was used for poly(lactic acid) fabrics dyeing with the disperse dye. The dyeing solution contained disperse dye (1% on the weight of fabric (owf)) and polyester (0.5 g/L) and a liquor ratio of 20:1 (pH 4.5; temperature and time: 110 °C × 30 min). The reflectance at the wavelength of maximum dye absorption was measured using an ACS spectrophotometer (Datacolor Comp., Lawrenceville, NJ, USA) to calculate the values of K/S (where K is the absorption coefficient and S is the scattering coefficient), an indirect means of analyzing the dye depth based on reflectance values for the color depth of the fabric. K/S is a function of the reflectance R, each sample was read in four different areas and the average value was recorded. All samples were read from the back of the fabric for consistency.

## 3. Results and Discussion

### 3.1. Structural Analysis

This series of compounds were made using chitosan, siloxane, and polyethylene glycol as the main raw materials. First, chitosan acetic acid was synthesized using chitosan and acetic acid, and then succinic anhydride and catalytic isopropoxide were added to form the Step 1 product. To increase the hydrophilicity of the hydrophobic siloxane, it was grafted with polyethylene glycol to synthesize the Step 2 product. Finally, the two products were combined into a series of chitosan derivative biological polyesters. The products PEG2000, PEG4000, PEG6000 and PEG8000 had yields of 52.87%, 67.09%, 67.74%, and 74.64%, respectively.

FTIR spectroscopy produces an infrared absorption spectrum by the absorption of vibrational and rotational energy levels by the molecule. It is used for the identification of functional groups. All molecules have a fixed energy; their bond stretching and bending lead to vibrations in other molecules. However, a functional group of a fixed molecule can only bend or vibrate at a specific frequency. When the molecule is exposed to infrared light, energy can only be absorbed when the frequency of the light is the same as the vibrational frequency of the element.

**Figure 1:**
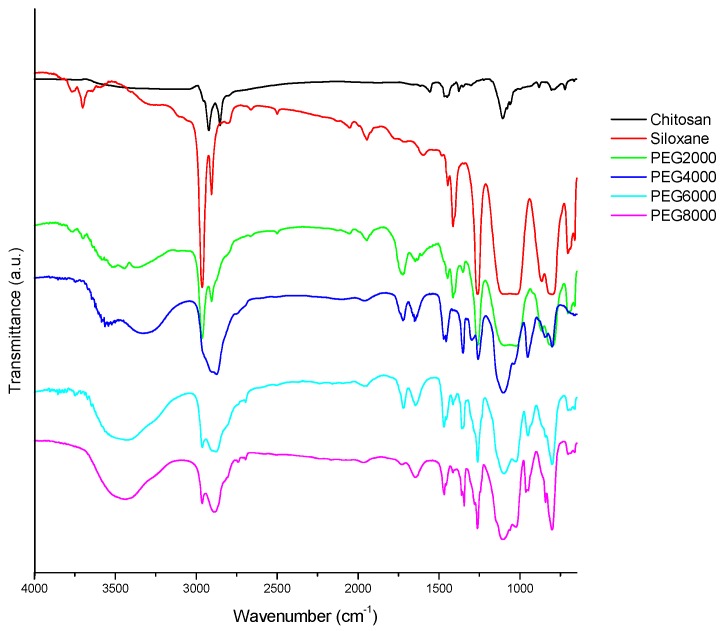
FTIR (Fourier Transform Infrared Spectroscopy) spectrum of chitosan derivative polyester; Chitosan: raw material; Siloxane: raw material; PEG2000: Chitosan/siloxane polyesters with PEG 2000 chain lengths.

In Figure 1, the characteristic absorption peaks corresponding to the various functional groups can be observed. Among them, the 810 cm^−1^ stretch is characteristic of Si-O. The peak at 1105 cm^−1^ signals the presence of C-O. Asymmetric vibration of -CH_2_ is evident at 2866 cm^−1^, and -OH stretching is apparent at 3450 cm^−1^. An asymmetric telomer absorption and asymmetric absorption occur at 2886 and 2966 cm^−1^, respectively. CH_3_ stretching absorption occurs with hydroxyl-OH stretching vibration absorption at 3450 cm^−1^. Chitosan has no absorption at 2886 and 2966 cm^−1^ because of its hydrophilicity, but hydrophobic siloxane has a strong signal at 2966 cm^−1^, signifying many -CH_3_ groups. It can been seen in Figure 1 that the Step 1 amide groups have been prepared; the peaks at 1650 and 1540 cm^−1^ were ascribed to the characteristic absorbance of amide groups. The successful preparation of the chitosan/siloxane polyesters was proven through the appearance of Step 3 ester bonds of product at 1730 cm^−1^.

### 3.2. Scanning Electron Microscopy

SEM images will provide information about the morphology of the treated fibers’ surface. Tungsten is heated to create light source that emits an electron beam, which is focused through a Wehne Cylinder to form a point light source a few tens of micrometers across. Under the accelerating voltage of the anode, it passes through the electrooptical system composed of an electromagnetic lens, which converges it into a small electron beam that is then focused on the surface of the test piece. Because of the scanning coil in the final lens, the interaction of high-energy electrons and the target material produces a variety of signals such as secondary electrons, backscattered electrons, absorbed electrons, transmitted electrons, X-rays, and cathode luminescence. The signal is received by the appropriate detector and amplified before it is sent to the image tube. The sample was cut into a square 0.3 cm across. A piece of double-sided adhesive was placed on the round metal stage, and then the sample was placed on the adhesive. An ion coater to coat the sample with platinum was used. The plated sample was placed under the scanning electron microscope, which took photos at different magnifications (under a vacuum).

**Figure 2:**
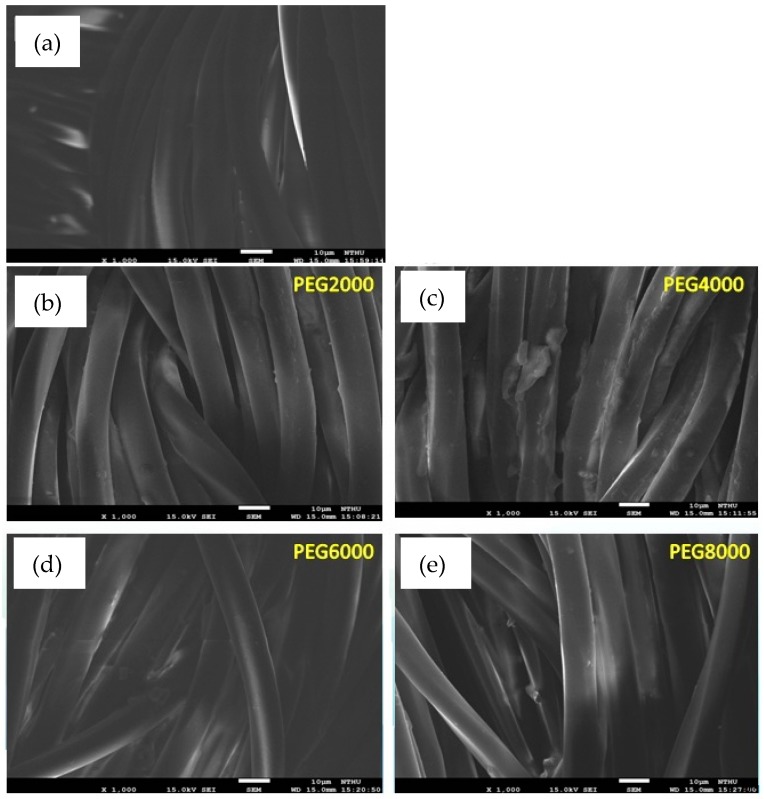
SEM (Scanning Electron Microscopy) images of poly(lactic acid) fabric uncoated and coated with chitosan/siloxane polyesters, magnified 1000 times; (**a**) uncoated; (**b**) coated Chitosan/siloxane polyesters with PEG 2000; (**c**) coated Chitosan/siloxane polyesters with PEG 4000; (**d**) coated Chitosan/siloxane polyesters with PEG 6000; (**e**) coated Chitosan/siloxane polyesters with PEG 8000.

Figure 2 shows the uncoated and coated poly(lactic acid) fabric under a scanning electron microscope. The surface of fibers before coating had not filled or sealed all of the inter-monofilament pores. Evidently, the surface of PEG4000 is coated unevenly, showing that the surfaces of some fibers were completely coated with the chitosan/siloxane polyesters and the smooth surface of the poly(lactic acid) fabric was hidden. It was supposed that the argon plasma treatment changed the poly(lactic acid) fabric surface and enhanced the deposition efficiency of chitosan/siloxane polyesters on the fabric surface. However, PEG8000 modified by plasma had better surface smoothness and uniformity.

### 3.3. High Resolution X-ray Photoelectron Spectrometer

HRXPS is generally used to detect the electron binding energy of an element, which can then be used to determine an unknown element. It uses high-voltage high-speed electrons to bombard aluminum or magnesium torelease X-rays. When X-rays hit the sample, the photocurrent can be used to determine the electron binding energy.

**Figure 3:**
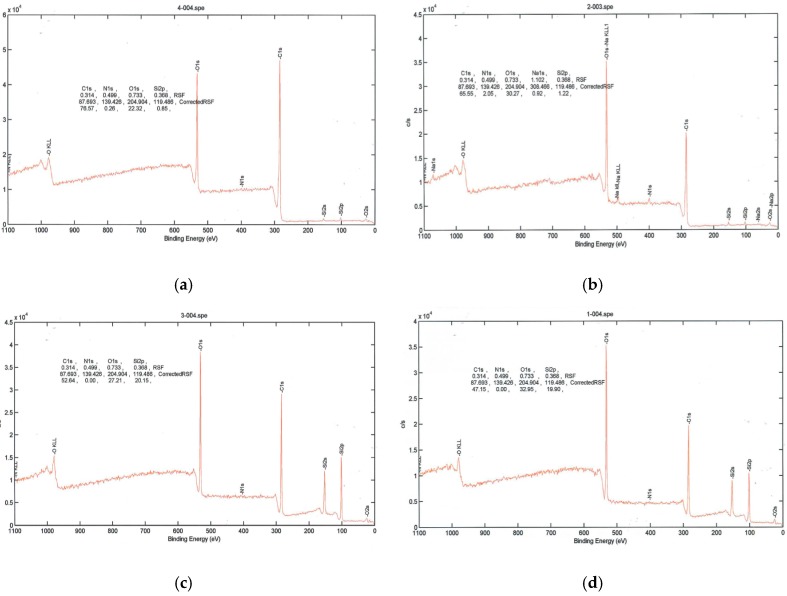
High-resolution X-ray photoelectron spectra. (**a**) PLA (Blank); (**b**) PLA surface plasma treatment; (**c**) PLA coated with PEG2000; (**d**) PLA coated with PEG2000 and then treated with plasma.

As seen in Figure 3, PEG2000-coated poly(lactic acid) fabric had silicon and increased amounts of oxygen after plasma treatment, while the amount of carbon decreased. When poly(lactic acid) fabric was treated with argon plasma, the surface atomic composition of carbon element decreased and the oxygen atomic component increased evidently. Furthermore, the oxygen on the surface of the control sample significantly increased from 22.32 to 32.95. The result indicated that many oxygen atoms or oxygen containing functional groups were introduced onto the poly(lactic acid) fabric by argon plasma, which significantly improved the hydrophilic property of the poly(lactic acid) fabric.

### 3.4. Antibacterial Properties

*S. aureus* had an OD value of 600 nm = 1.8; CFU = 1 × 10^9^. Table 1 shows the total number of colonies on the poly(lactic acid) fabric (uncoated and coated with PEG2000) after 24 h. Table 1 shows that the poly(lactic acid) fabric inhibits bacterial growth. The control and the poly(lactic acid) fabric that had not undergone plasma treatment had fewer colonies, while the fabric coated with chitosan derivative polyester did not seem to have high efficacy. This could be due to the lack of antibacterial properties by the cloth or the effect of dilution. The chitosan/siloxane polyesters were cationic-nonionic compounds containing a nonionic hydrophilic polyoxyethylene moiety in structure. It is generally accepted, however, that the presence of polyoxyethylene chain structures improves the industrial applicability of nonionic compound. Because the chitosan/siloxane polyesters possessed polyoxyethylene chain, its structure produced hydrogen bond in the solutions, providing more solubility. Enhancing the hydrophilicity of compounds (by increasing their oxyethylene chain length) usually leads to a decrease in antibacterial activities, i.e., increasing the length of the polyethylene chain resulted in a decrease in the antibacterial properties.

### 3.5. Dyeing Properties

PLA’s green credentials stem from the fact that the lactic acid used in its manufacture is derived from fermentation of vegetable matter. In addition to rising customer demands for biodegradable products with low toxicity playing a role in the growing popularity of the material, increasing environmental awareness and the introduction of legislation is forcing industry to seek more ecologically friendly materials such as PLA. Some properties of PLA cause difficulties in textile application. First, PLA hydrolyzes more easily than other synthetic polyesters, leading to strength and elongation losses. Secondly, dye selections for PLA are only limited in popularity or energy levels of commercial disperse dyes [22].

One of the important indicators of dyeing performance is the depth of the dye. The Kubelka–Munk dyeing depth equation establishes a relationship among the absorption coefficient (K), the scattering coefficient (S) of the measured object, and the concentration (C) of the colored substance in the sample. The higher is the K/S value, the darker is the sample, the higher is the concentration of the colored material, and the better the dyeing performance. Polyester can be used as a wetting agent, a leveling agent, a solubilizing agent, and a precipitation-preventing agent. Hence, the interaction of the dye with the polyester is very important in many dyeing processes such as fabric dyeing, photo printing, and inkjet printing.

CIB LAB is based on the theory that a color cannot be both green and red, nor can it be both blue and yellow. Therefore, single values can be used to describe red and green, and yellow and blue. The CIB LAB tolerance formula centers on the standard and gives the individual L*, a*, and b* values with a ± error range.

(1) $$\Delta E=\sqrt{{\left(\Delta L^{*}\right)}^{2}+{\left(\Delta a^{*}\right)}^{2}+{\left(\Delta b^{*}\right)}^{2}}$$

(2) ΔL* = L*sample − L*standard (difference in brightness; +: shallow)

(3) △a* = a*sample − a*standard (+: reddish; −: greenish)

(4) △b* = b*sample − b*standard (+: yellowish; −: bluish)

Table 2 shows the degree of dyeing. The first column from the left, seventh row (+PEG2000) displays the names of the poly(lactic acid) fabric coated with chitosan derivative polyester that underwent surface plasma treatment. The interaction between the additive and the dye is shown in Table 2. Larger K/S values indicate better dyeing rate. Table 2 indicates that the surface plasma treatment imparted a higher K/S values than before surface plasma treatment, which means we the surface plasma treatment imparted excellent dyeing efficiency for the poly(lactic acid) fabric. In addition, we can see in the table that, after the surface plasma treatment, the longer PEG chain lengths led to higher the K/S values. PEG8000 without plasma treatment produced a K/S value of 1.645, but the K/S value of +PEG8000 became 1.933 after the plasma treatment. Therefore, we can conclude that plasma treatment can increase the effect of dyeing of the poly(lactic acid) fabric. When the PEG chain length increases, the ΔE value becomes smaller after the plasma treatment. Untreated PEG8000 had a ΔE value of 3.72, which became 1.92 after surface plasma treatment. Smaller ΔE values result in more even dyeing; thus, the plasma treatment has a positive effect. It is reasonably deduced that argon plasma treatment introduced polar oxygen groups and improved the hydrophilicity of fiber surface. As already shown in SEM observation, a rough surface with uniformly distributed micro pits was created by plasma treatment. Therefore, the rough surface with hydrophilic polar groups led to the enhancement of the absorbability and deep penetration of the dyestuff cover around chitosan/siloxane polyesters with hydrophilic end groups into the fiber surface [23,24,25]. Thus, the bonding and retaining of the dyestuff molecules were increased for plasma treated poly(lactic acid) surface.

## 4. Conclusions

This study increased the numbers of hydrophilic OH groups on chitosan and the hydrophobic CH_3_ groups on siloxane to extend the hydrophilic and hydrophobic ends. These were made into a series of chitosan-derived biological polyesters. Different functional groups were introduced into the molecular structure of the chitosan derivative polyester, and its hydrophilic groups were linked to the hydrophobic chains of the poly(lactic acid) fiber; thus, the chitosan remained fixed to the poly(lactic acid) fiber and did not detach. Argon plasma treatment was used to significantly increase the surface area of the fabric, so that the surface would be more hydrophilic. The oxygen content of the poly(lactic acid) fabric increased after treatment. Plasma treatment increased the hydrophilicity of the surface. However, the antibacterial test did not show obvious effects of plasma-treated poly(lactic acid) fiber in inhibiting bacterial growth. Coating PEG2000 on the fabric did not show any effect as well. Presumably, it was not released by the cloth, or the dilution affected its antibacterial properties. Plasma-treated +PEG8000 poly(lactic acid) fabric with dispersed red dye had the greatest K/S value, showing that it has the best degree of dyeing. It also had the smallest ∆E, signifying even dyeing.

## Figures and Tables

**Table 1:** Colonies on control, poly(lactic acid) fabric, and PEG2000-coated poly(lactic acid) fabric.

Condition	CFU/mL		
	Control	PLA	PLA + PEG2000
dilution	2.8 × 10^4^ ± 0.33	2.3 × 10^4^ ± 0.31	2.3 × 10^4^ ± 0.41
dilution and surface plasma treatment	2.6 × 10^4^ ± 0.24	2.2 × 10^4^ ± 0.57	1.8 × 10^4^ ± 0.22

**Table 2:** Dyeing properties of the dispersed red dye.

Sample	ΔL	Δa	Δb	K/S	ΔE
R/Standard	Standard	Standard	Standard	Standard	Standard
PEG2000	2.13	2.16	0.33	1.700 ± 0.03	3.05 ± 0.06
PEG4000	1.05	0.17	0.21	1.929 ± 0.05	1.09 ± 0.12
PEG6000	2.10	2.00	0.71	1.715 ± 0.11	2.98 ± 0.09
PEG8000	2.20	2.94	0.57	1.645 ± 0.20	3.72 ± 0.14
+PEG2000	3.84	4.39	1.07	1.397 ± 0.24	5.93 ± 0.17
+PEG4000	3.12	3.99	0.91	1.471 ± 0.09	5.15 ± 0.22
+PEG6000	1.78	1.82	0.59	1.776 ± 0.14	2.61 ± 0.04
+PEG8000	1.43	0.80	1.00	1.933 ± 0.07	1.92 ± 0.14
